# Radial vs. Dorsal Approach for Elastic Stable Internal Nailing in Pediatric Radius Fractures—A 10 Year Review

**DOI:** 10.3390/jcm11154478

**Published:** 2022-07-31

**Authors:** Raffael Cintean, Alexander Eickhoff, Carlos Pankratz, Beatrice Strauss, Florian Gebhard, Konrad Schütze

**Affiliations:** Department of Trauma-, Hand-, and Reconstructive Surgery, Ulm University, Albert-Einstein-Allee 23, 89081 Ulm, Germany; alexander.eickhoff@uniklinik-ulm.de (A.E.); carlos.pankratz@uniklinik-ulm.de (C.P.); beatrice.strauss@uniklinik-ulm.de (B.S.); florian.gebhard@uniklinik-ulm.de (F.G.); konrad.schuetze@uniklinik-ulm.de (K.S.)

**Keywords:** ESIN, forearm fractures, EPL, SBRN, nerve damage, tendon lesion, approach, radial fracture, pediatric

## Abstract

Background: Forearm fractures are one of the most common fractures in children. Over the last years, a tendency towards surgical treatment was seen, especially closed reduction and internal fixation with elastic stable internal nailing (ESIN). Despite an overall low complication rate being described, a risk of intraoperative complications remains. Material and Methods: A total of 237 patients (mean age 8.3 ± 3.4 (1–16) years) with forearm or radius fractures treated with ESIN between 2010 and 2020 were included in the study. The retrospective review of 245 focused on fracture pattern, pre- and postoperative fracture angulation, intra- and postoperative complications, and surgical approach for nail implant. The fracture pattern and pre- and postoperative angulation were measured radiographically. Complications such as ruptures of the extensor pollicis longus (EPL) tendon and sensibility disorders of the superficial radial nerve were further analyzed. Results: In 201 cases (82%), we performed a dorsal approach; 44 fractures (17.9%) were treated with a radial approach. In total, we found 25 (10%) surgery-related complications, of which 21 (8.6%) needed further surgical treatment. In total, we had 14 EPL ruptures (5.7%), 4 sensibility disorders of the superficial radial nerve (1.6%), 2 refractures after implant removal (0.8%), 2 superficial wound infections (0.8%), and 1 child with limited range of motion after surgery (0.4%). No statistical significance between pre- and postoperative angulation correlated to fracture patterns or diameter of the elastic nail was seen. As expected, there was a significant improvement of postoperative angulation. Using radial approach in distal radial fractures showed a lower rate of surgical related complications, 2.3% of which need further surgical treatment as well as better postoperative angulations compared to the dorsal approach (8.5%). Conclusion: Especially due to the low risk of damaging the EPL tendon, the radial approach showed a lower complication rate which needed further surgical treatment. The risk of lesions of the superficial radial nerve remains.

## 1. Introduction

With an incidence of approximately 1 in 100 children per year, forearm fractures are one of the most common fractures in children. Among these injuries, diaphyseal forearm fractures are the third most common long-bone fractures in children [1,2]. Most pediatric fractures have a greater remodeling potential. Due to this fact, nonoperative treatment can be performed in most pediatric nondisplaced or minimally displaced radial and ulnar shaft fractures [3]. Despite this, the operative treatment with closed reduction and intramedullary nailing due to the minimally invasive approach gained popularity in the last 20 years. Cheng et al. reviewed the treatment of pediatric forearm fractures at their institution in the time frame of 10 years between 1985 and 1995 and found a trend towards intramedullary stabilization from 1.8% to 22% as an alternative to closed reduction and cast immobilization in pediatric forearm fractures [4]. Even though literature shows that intramedullary nailing has a generally low complication rate, a risk of intra- and postoperative complications remains. Different studies have shown a complication rate up to 67%. Kruppa et al. reported a complication rate of 8.9% in 202 patients [5], Smith et al. reported a complication rate of 42% [6], whereas other researchers did not report any complications after surgery [7]. Concerning complications in treatment of pediatric forearm fractures, rupture of EPL tendon and lesion of the superficial radial nerve are mentioned. Therefore, controversy exists regarding the preferred entry point for intramedullary nailing at the distal radius [8]. A dorsal approach through the Lister tubercle avoids the possible risk of damaging the superficial radial nerve but sets the EPL tendon at risk for injury [9]. Although studies describe the different approaches and the consecutive risks, a clinical comparative study could not be found in the literature search.

This study was performed to investigate and compare the advantages and disadvantages of both approaches to the distal radius using ESIN.

## 2. Methods

The study was a retrospective exploratory review at a Level One Trauma Center. Between January 2010 and December 2019, patients with forearm fractures were identified using the ICD-Code (International Statistical Classification of Diseases and Related Health Problems) as well as the OPS Code, which is the German equivalent to the International Classification of Procedures in Medicine (ICPM). We included every patient under 16 years of age treated with open or closed reduction and internal fixation with at least one radial intramedullary nail. Patients with previous fractures of one forearm were included provided there was adequate trauma, no osteosynthesis material was implanted, and the previous fracture was thought to have no influence on the new fracture. Patients with nonoperative treatment, other osteosynthesis techniques, and patients with multiple injuries, pathological fractures, or pre-existing bony conditions (i.e., osteogenesis imperfecta) were excluded. Patients with implanted osteosynthesis material in the fractured arm were excluded. A total of 245 fractures in 237 patients, surgically treated using at least one radial ESIN, were included. All found complications were chart reviewed and categorized.

The study was approved by the institutional ethical committee (89/20-FSt/TR).

### 2.1. Angulation

All patient charts as well as the pre- and postoperative radiographic data were reviewed by two experienced attending surgeons. The fracture patterns were analyzed on available anterior–posterior as well as lateral performed X-rays. The pre- and postoperative angulations of the radius in both plane anterior–posterior and lateral imaging were measured in the institutional PACS system and categorized in 3 groups with 0 degree pre- and postoperative angulation, between 1 and 5 as well as above 5 degrees in anterior-posterior and lateral plane. The radial diaphysis was subdivided in proximal, medial, and distal thirds for statistical reasons.

### 2.2. Operative Technique

In all patients, we used the Synthes Titanium Elastic Nail System (West Chester, PA, USA) with diameters of 1.5 mm up to 3.0 mm, generally to be up to 2/3 of the diameter of the intramedullary canal. The ESIN is usually prebend to secure an intramedullary 3-point-fixation. The ulnar ESIN was implanted through a 3 to 5 mm incision at the olecranon distal of the growth plate. The radius was either stabilized through a 3 to 5 mm distal dorsal incision near the Lister tubercle or a distal radial approach proximal of the growth plate was chosen also through a 3 to 5 mm radial incision. The approach as well as the used osteosynthesis was chosen by the attending surgeon’s choice by case. During all surgeries, free range of motion (ROM) of the elbow and wrist was tested after stabilization. All surgeries were performed by or under supervision of an experienced attending surgeon.

Postoperatively, the patients were immobilized in a long arm cast for forearm fractures and in a short arm cast only for radial fractures for the first 2–4 days after surgery. Longer cast immobilization was performed individually depending on the intraoperative fracture stability and the surgeon’s decision. As it is institutional practice, an anterior–posterior as well as lateral X-ray was performed on the day after the surgery. Most patients were discharged the next day, the mean hospitalization time was 1.69 days (range 1–3 days). Clinical and radiographic follow-up was performed 6 and 12 weeks after surgery if no complication occurred.

### 2.3. Complications

Complications such as lesions of the superficial branch of the radial nerve (SBRN), ruptures of the extensor pollicis longus tendon, wound infections, postoperative hematoma, refracture after implant removal, restricted mobility after surgery, and lesions of the ulnar and median nerve were recorded. Complications, especially ruptures of the EPL tendon and lesions of the superficial radial nerve, were further analyzed regarding fracture type, surgical treatment, treatment of complication, and final outcome.

### 2.4. Statistics

Data analysis was performed with IBM SPSS Statistics (V12.0, IBM, Armonk, NY, USA) and Microsoft Excel (V15.2, Microsoft, Redmond, WC, USA). Demographic characteristics were described as mean and standard deviation. Logistic regression was performed for primary outcome measures considering all variables related to the pre- and postoperative angulation and postoperative complications.

## 3. Results

The average age of our patients was 8.3 ± 3.4 years (range 1–16 years). We found 202 radial and ulnar fractures and 43 radial fractures. In the radiographic analysis of the cohort, we found 182 middle diaphyseal fractures, 23 proximal diaphyseal fractures, and 40 distal diaphyseal fractures treated with ESIN. Three patients presented with a Gustilo–Anderson type I open fracture which needed extended wound debridement [10].

In all 202 cases with radial and ulnar fractures, we performed intramedullary nailing of both radius and ulna; in 46 cases only, the radius was reduced and stabilized. The mean time of surgery was 27.6 ± 12.5 min with a range from 8 to 82 min. In 201 cases, the dorsal approach through the Lister tubercle was performed. After showing a rising incidence of EPL lesions, a change of practice in the institution was done. After that, 44 fractures were operated with a radial approach.

Demographic factors such as age, sex, and side of fracture showed no statistical significance correlated to postoperative angulation or complications. Intraoperative surgical factors such as thickness of the used elastic nail or operative time did not show statistical significance to postoperative angulation or complications.

The follow-up averaged 4.7 ± 4.2 months (range 1.7–28.1 months). Implant removal was performed on average after 3.3 ± 2.8 months (range 1.1–11 months) after clinical and radiographic healing was ensured. A total of 23 patients (9.2%) were lost during follow-up.

### 3.1. Angulation

**Figure 1:**
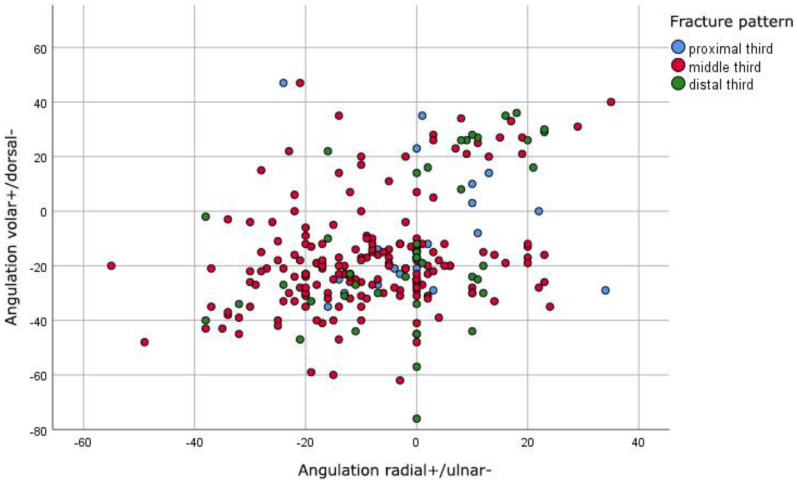
Preoperative distribution of angulations in degrees of fractures in anterior-posterior (x axis) and lateral (y axis) plane.

In the anterior–posterior plane, the fracture angulation of the radius distributed between 70 radial angulations with a maximum of 35 degrees and 149 ulnar angulations with a maximum of 55 degrees. There was no significant angulation in the anterior–posterior plane of 30 fractures. In the lateral plane, we found 196 dorsal angulations with a maximum of 76 degrees and 46 volar angulations with a maximum of 47 degrees. Three fractures showed no significant angulation in the lateral view. As shown in Figure 1, most fractures were dorsal and ulnar angulated (Figure 1).

**Figure 2:**
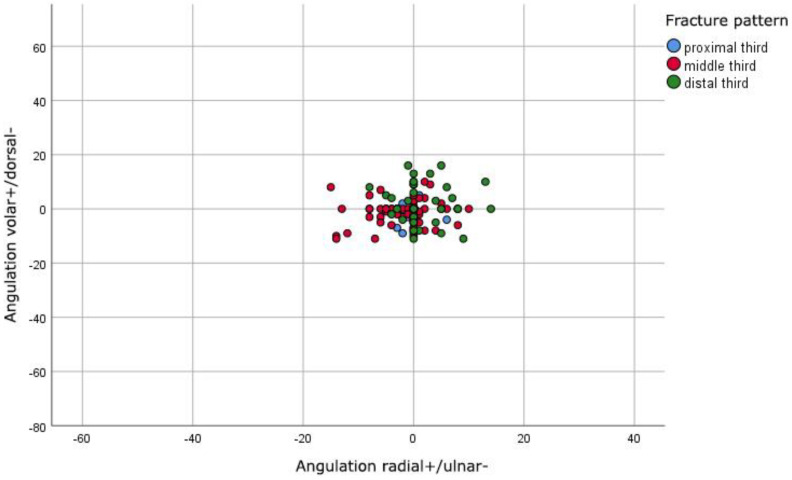
Postoperative distribution of angulations in degrees of fractures in anterior–posterior (x axis) and lateral (y axis) plane.

**Figure 3:**
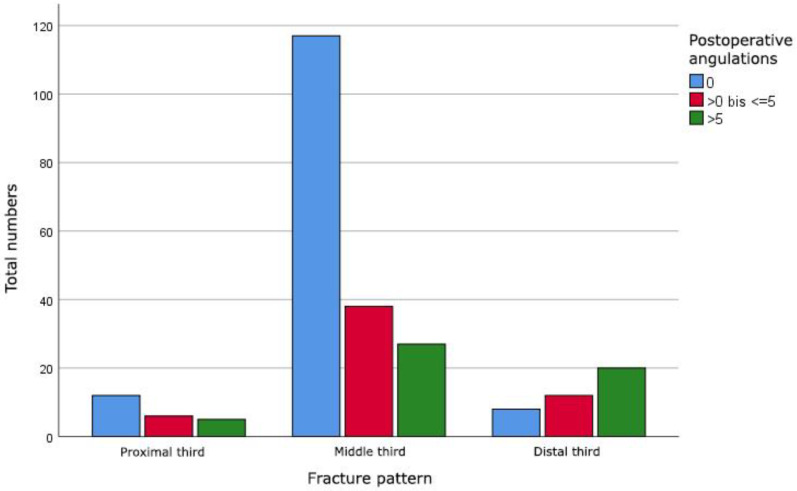
Comparison of fracture pattern and maximal postoperative angulations of diaphyseal radial fractures in degrees in both planes.

The postoperative radiographic measurements showed an expected improvement of the angulations, even though angulations up to 18 degrees in all planes could be found (Figure 2). In relation to the surgical approach, no significant difference (*p* = 0.129) was seen. However, we found a significant correlation (*p* < 0.001) between the fracture pattern and the postoperative angulations. Fractures in the distal diaphysis showed a significantly higher postoperative angulation compared to fractures in the proximal and middle diaphysis (Table 1, Figure 3).

**Figure 4:**
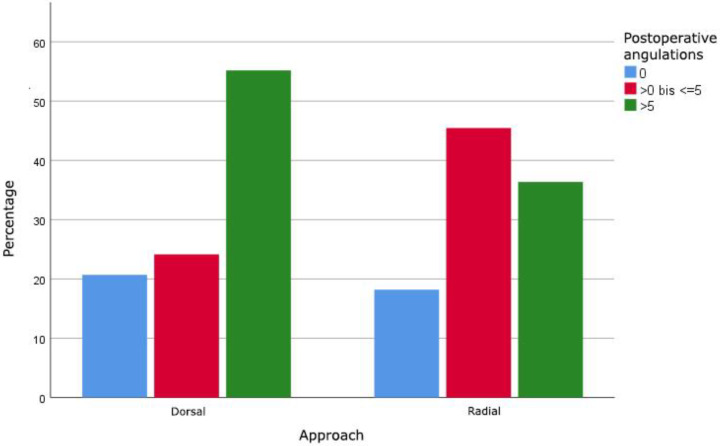
Maximal postoperative angulations in degrees in both planes of distal radial diaphysis fractures operated with dorsal or radial approach.

In 29 out of 40 fractures of the distal third of the diaphysis, the ESIN was performed through a dorsal approach, 11 fractures were operated on using a radial approach. No significant correlation (*p* = 0.414) between the approach and postoperative angulation of distal forearm and radial fractures was shown. A tendency towards the radial approach in terms of better maximal postoperative angulation in both planes and radiographic outcome was found (Figure 4).

### 3.2. Complications

In total, 25 (10.0%) surgery-related complications occurred in the study population. A total of 21 (8.6%) children required further surgical treatment (Table 2).

A total of 16 (7.9% of dorsal approaches) ruptures of the extensor pollicis longus tendon were observed and required operative treatment, one child showed an entrapment of the EPL tendon and did not need further treatment after surgical release. In one case, an EPL rupture due to adhesion to the callus formation was observed. One child with EPL rupture was lost during follow-up. The mean time from initial surgery to EPL repair was 3.3 ± 2.4 months (range 21 days–6.3 months). All EPL repairs were treated with a transfer of the extensor indicis proprius (EIP) tendon. A significance (*p* < 0.001) between the chosen approach for ESIN and EPL rupture was found. All of them were operated on using a dorsal approach. No significant correlation between diameter (range 1.5–3.0 mm) of the ESIN and EPL rupture could be found. All children showed good outcome after EPL repair in the clinical follow-ups.

In 4 (9.1% of radial approaches) cases, we observed hypesthesia of the thumb due to lesion of the superficial branch of the radial nerve (SRNB) after ESIN. In all cases, a radial approach with a skin incision of around 5 to 8 mm was performed. Further surgical treatment was required in none of the cases, as sensibility improved over time. After early implant removal and cast immobilization after 1.1 months, one child complained about a persistent mild sensibility disorder which was addressed with physiotherapy. The palsy was consistent after a follow-up of 6 months after ESIN removal. No further surgical treatment was required.

Two (0.8%) refractures occurred due to new adequate trauma at 1.1 and 1.6 months after implant removal. In one case, we decided to perform open reduction and plate osteosynthesis. In one case, a secondary loss of reduction 21 days after ESIN implantation was observed which required early implant removal, open reduction, and plate fixation. No mal- or nonunion occurred.

Two superficial wound infections were observed and required surgical treatment. No compartment syndrome occurred due to trauma or surgery.

One child with a diaphyseal forearm fracture showed limited range of motion (ROM) in supination of >20° after radial and ulnar ESIN implantation. After hardware removal, the ROM improved to almost normal.

Implant removal was performed on average after 3.3 ± 2.8 months (range 1.1–11 months) after clinical and radiographic healing was ensured. Except the 23 patients lost during follow-up, all children got implant removal at our department.

## 4. Discussion

In this study, both approaches were shown to have unique complications described in the literature. However, it was found that the radial approach to the distal radius was associated with significantly fewer complications that required further surgical revision. Especially for the critical distal diaphyseal fractures, we could additionally demonstrate a better reduction in the radiographic controls.

Fixation with ESIN is already a standard operative technique for both or single bone forearm fractures in children providing a primary definitive treatment [6,11,12,13]. However, in the literature there is no clear opinion on approaches to the radius for ESIN. Many authors perform the dorsal approach through the Lister‘s tubercle [7,14,15]. As initially described by Lascombes et al., a radial approach to the distal radius can be performed [15,16].

Although low complication rates were generally reported, a large diversity of complication rates from 8.9% up to 67% are reported in the literature [5,7,11,14,17].

In our study, we included 237 patients with 245 fractures of the forearm treated with at least one radial ESIN with an overall surgery related complication rate of 10%. Focusing on the dorsal approach, we found 16 (7.9% of dorsal approaches, 6.5% in total) ruptures of the EPL tendon, which all occurred using a dorsal approach. Flynn et al. reported EPL injuries from ESIN after dorsal approach in 2 of 103 patients (1.9%) [14]. In a small cohort of 17 patients, Lee et al. reported 3 EPL ruptures after surgical treatment with ESIN and a dorsal approach of pediatric forearm fractures (17.6%) [15]. A recent systematic review by Murphy et al. listed a total of 30 patients with EPL ruptures after ESIN of forearm fractures in 338 children, all using the dorsal approach (8.9%). In their study, the EPL lesion is described as unique complication of the dorsal entry approach [18]. We had similar findings in the present study with all EPL lesions being associated with dorsal approaches. It is recommended when performing the dorsal approach to use a mini-open incision to avoid lesions of the EPL tendon via effective retraction. Varga et al. even recommended intraoperative visualization of the EPL using sonography to avoid lesion of the tendon, though mentioning basic skills in sonography are necessary [19]. Even though in the literature most surgeons use the mini-open technique to avoid damaging the tendon, a high risk of EPL tendon ruptures remains [14,18,20].

Although seeming safer, the radial approach has been associated with complications [16]. Fernandez et al. reported lesions of the superficial branch of the radial nerve in 15 out of 553 patients after implantation or removal of ESIN (2.7%). In 2 patients, it was regressive but persistent. No further surgical treatment was necessary [17]. Lyman et al. reported 3 superficial radial nerve palsies in 456 patients after ESIN using the radial approach (0.9%). No further surgical treatment was required [15]. Schmittenbecher et al. found 9 lesions of the SBRN in 300 patients (3%) [21]. After showing a rising incidence of EPL ruptures, a change of practice in our institution to radial approach was indicated. We performed the radial approach to the distal radius in 44 cases. We found 4 lesions of the SBRN after radial approach (9%). In one case, the sensibility disorder was regressive but consistent after a follow-up of 6 months. No cases required further surgical treatment. Different studies recommend the radial approach using a mini-open incision to ensure the visualization and effective retraction of the superficial branch of the radial nerve [1,9,17,21]. We considered the lesion of the SBRN a less severe complication not requiring further surgical treatment.

Unrelated to approach, refracture after implant removal showed the third highest complication rate in the present study with 0.8%. All patients with refractures showed an adequate trauma. Similar or higher numbers can be found in the literature. Fernandez et al. reported 13 refractures in 537 patients after implant removal within 2 years (2.4%) [17]. Kruppa et al. reported 7 refractures after ESIN removal in 202 cases (3.5%) [5]. As a main reason for refractures early implant removal is mentioned [9,15,17,21]. We removed ESIN after an average of 3.3 months after clinical and radiographic 4-cortices healing. Literature recommends longer times for implant removal of 6 to 12 months [7,9,15,16,17]. Due to low incidence in the present study, no statistical correlation between implant removal and refracture could be found. Other authors describe a higher refracture risk after early implant removal [21,22,23].

The pre- and postoperative angulation showed the expected improvement in all planes. Focusing on the distal diaphyseal fractures, a better postoperative outcome of the maximal angulation in both planes after radial approaches was found. The literature recommends K wire fixation for pediatric metaphyseal or epiphyseal fractures but highlights the difficulties in distal diaphyseal fractures with K wire fixation [22,24,25]. No clear guidelines exist for distal diaphyseal or metaphyseal fractures. Du et al. used anterograde ESIN as it is mentioned that there is not enough space to secure a stable fixation in the distal fragment [24]. We used the same technique in the past. In this study, we found 40 distal diaphyseal fractures treated with retrograde ESIN and found good results in clinical and radiographic outcome. Despite being reported not to be ideal for fractures in distal diaphyseal or metaphyseal fractures, the radial approach showed better results in maximal postoperative angulation in both planes (Figure 4). Together with this and our findings of less complications which need further surgical treatment, we recommend the radial approach for ESIN in most diaphyseal pediatric fractures.

The study shows several limitations. The retrospective aspect of the study comes with its inherent problems. Due to the late change of surgical technique, we only had 44 cases of radial approaches to the distal radius. More cases would provide better data for statistical significance. Due to the wide age range of the included patients, differences between bone healing and maximal angulation may occur. Moreover, some patients may have been missed because of errors in coding.

## 5. Conclusions

We recommend taking the radial approach into consideration for most radial diaphyseal fractures due to lower complication rate which require further surgical treatment. Care should be taken during nail insertion and removal to avoid damage to the SBRN. Removal can be performed after 3 months and clinical and radiographic healing.

## Figures and Tables

**Table 1:** Fracture pattern compared to postoperative angulations.

Postoperative Angulation		Fracture Pattern			Total
		Proximal Third	Middle Third	Distal Third	
0°	N	12	117	8	137
	Percentage	52.2%	64.3%	20.0%	55.9%
>0° to ≤5°	N	6	38	12	56
	Percentage	26.1%	20.9%	30.0%	22.9%
>5°	N	5	27	20	52
	Percentage	21.7%	14.8%	50.0%	21.2%
Total	N	23	182	40	245
	Percentage	100.0%	100.0%	100.0%	100.0%

**Table 2:** Surgery related complications (EPL = extensor pollicis longus, SBRN = superficial branch of radial nerve, ESIN = elastic stable intramedullary nailing, ROM = range of motion.

Surgery-Related Complications	Total (%)	Dorsal Approach	Radial Approach
EPL rupture	16 (6.5%)	16 (7.9%)	0
Lesion of SBRN	4 (1.6%)	0	4 (9.1%)
Refracture after ESIN removal	2 (0.8%)	1 (0.5%)	1 (2.2%)
Wound infection	2 (0.8%)	2 (1%)	0
Limited ROM	1 (0.4%)	1 (0.5%)	0
Total	25 (10%)	20 (9.9%)	5 (11.4%)
